# Low levels of soluble DPP4 among Saudis may have constituted a risk factor for MERS endemicity

**DOI:** 10.1371/journal.pone.0266603

**Published:** 2022-04-12

**Authors:** Khaled R. Alkharsah, Salma Ali Aljaroodi, Jawad Ur Rahman, Awatif N. Alnafie, Reem Al Dossary, Reem Y. Aljindan, Amani M. Alnimr, Jamal Hussen

**Affiliations:** 1 Department of Microbiology, College of Medicine, Imam Abdulrahman Bin Faisal University (IAU), Dammam, Saudi Arabia; 2 Department of Pathology, College of Medicine, King Fahad Hospital of the University, Imam Abdulrahman Bin Faisal University, Dammam, Saudi Arabia; 3 Department of Microbiology, College of Veterinary Medicine, King Faisal University, Al-Ahsa, Saudi Arabia; Taif University, SAUDI ARABIA

## Abstract

Most of the cases of Middle East respiratory syndrome coronavirus (MERS-CoV) were reported in Saudi Arabia. Dipeptidyl peptidase-4 (DPP4) was identified as the receptor for the virus. The level of soluble DPP4 (sDPP4) was found to be reduced in MERS-CoV infected patients while high levels of sDPP4 were suggested to be protective against MERS-CoV in animal models. We investigated whether the Saudi population has lower levels of sDPP4 which makes them more susceptible to MERS-CoV infection and, therefore, could explain the larger number of cases from the country. Blood samples were collected from 219 Saudi blood donors and 200 blood donors from other ethnic groups. The plasma level of sDPP4 was measured by ELISA and the following SNPs in the *DPP4* gene; rs35128070, rs1861978, rs79700168, and rs17574, were genotyped by TaqMan SNP genotyping assay. The average level of plasma sDDP4 was significantly lower in Saudis than other Arabs and non-Arabs (P value 0.0003 and 0.012, respectively). The genotypes AG of rs35128070 and GT of rs1861978 were significantly associated with lower sDPP4 among Saudis (P value 0.002 for each). While both genotypes AA and AG of rs79700168 and rs17574 were associated with significantly lower average sDPP4 level in Saudis compared to other ethnic groups (P value 0.031 and 0.032, and 0.027 and 0.014, respectively). Herein, we report that the Saudi population has lower levels of plasma sDPP4 than other ethnic groups, which is associated with genetic variants in the *DPP4* gene. This may have contributed to increase the susceptibility of the Saudi population to MERS-CoV infection and could be a factor in the long-lasting persistence of the virus in the country.

## Introduction

Dipeptidyl peptidase-4 (DPP4), also called CD26, is a hydrolase enzyme that cleaves two amino acids from oligopeptides or shorter peptides at proline or alanine residues from their N-termini [1]. This transmembrane protein is located on the cell surface of many epithelial and endothelial tissues in several organs such as kidneys, lungs, liver, and intestine as well as on immune cells including T cells, and activated natural killers and B cells [1]. The substrate of DPP4 is a wide range of pancreatic polypeptides, chemokines, neuropeptides, and peptides of the glucagon family [2]. In addition to its peptidase activity, DPP4 plays an important role in the development and stimulation of T cells though binding to adenosine deaminase (ADA), CD45, CXR-4, and Caveolin-1 [2]. Its expression levels and activity were found to be altered in psychological diseases, autoimmune diseases, inflammatory and infectious diseases, metabolic and cardiovascular disorders, and tumors [1, 3, 4]. DPP4 is also a recognized therapeutic target for type 2 diabetes mellitus (T2DM) through multiple inhibitors [5–7]. In addition to its transmembrane form, DPP4 is present in functional soluble form (sDPP4) in most body fluids including serum, urine, saliva, and cerebrospinal fluid (CSF) [8–10]. The levels of the sDPP4 were found to vary with different pathological conditions including infection [1, 9]. High levels of sDPP4 were associated with poor treatment prognosis in hepatitis C virus genotype 1 infection and with progression to liver fibrosis and cirrhosis [11–13]. In HIV infected individuals, the levels of sDPP4 were intensely reduced and were associated with rapid progression to AIDS [14]. Plasma levels of sDPP4 were also found to be reduced in patients hospitalized for severe COVID-19 infection [15].

DPP4 was identified as the receptor for the Middle East respiratory syndrome-coronavirus (MERS-CoV), which emerged in April 2012 [16, 17]. The receptor binding site of the spike (S) protein of the virus was found to bind to 14 amino acids between blades IV and V of DPP4 and initiates virus entry through receptor mediated endocytosis [18, 19]. Notably, the enzymatic activity of DPP4 was not required for MERS-CoV entry [20]. Facilitated by the ubiquity of DPP4 on several tissues, MERS-CoV could infect multiple organs which may eventually lead to multisystem failure and death with about 35% case fatality rate [21]. Until the end of May 2021, 2574 MERS-CoV cases were reported in 27 countries worldwide [21, 22]. However, 84.5% of the cases originated from Saudi Arabia [22]. The remainder cases were mainly transmitted from the Middle East to countries such as Tunisia, United Kingdom, France, Italy, and South Korea and were described as either sporadic cases or a focal cluster of cases that were contained later [21, 23]. The virus continued to spread in Saudi Arabia from 2012. The lengthy presence of the virus in Saudi Arabia could be attributed to the presence of the intermediate host or other genetic risk factors. The dromedary camels were identified as the intermediate host for the virus with risk of transmission to human [24–26]. Several reports have described the detection of MERS-CoV-like virus in dromedary camels from countries in the Middle East and Africa, however, no sustained transmission to human was reported in these counties [27–29]. Hence, the long period of virus persistence and transmission in Saudi Arabia could be influenced by other host specific related factors. Interestingly, levels of sDPP4 were found to be reduced in patients infected with MERS-CoV [30]. Additionally, meta-analysis studies showed that after treatment with DPP4 inhibitors, upper respiratory tract and urinary tract infections increased significantly [7, 31]. On the other hand, in a study using human DPP4 transgenic mice, higher levels of serum DPP4 were found to be protective against MERS-CoV infection with attenuated morbidity and reduced mortality [32].

In order to further understand the risk factors associated with acquiring infection by MERS-CoV, the current study investigated the possibility that Saudi population could have lower levels of sDPP4, which in turn constitutes a predisposing factor for MERS-CoV infection and may be driven by presence of genetic variants. We measured sDPP4 level in the plasma of Saudi individuals and individuals from other ethnic backgrounds and correlated these levels with single nucleotide polymorphisms (SNPs) in the *DPP4* gene assuming that these SNPs affect the expression and the levels of sDPP4.

## Materials and methods

### Samples and setting

Blood samples (3-5ml) were collected from volunteer blood donors attending the blood bank section of King Fahd Hospital of the University (KFHU), an academic 550-bed center in Al-Khobar at the Eastern Province of Saudi Arabia over a two-month period (December 2019-January 2020). Plasma was immediately separated from blood cells by centrifugation at 5000 rpm for 10 minutes. Both plasma and cell fractions from each sample were stored at -80°C till the time of analysis.

#### Inclusion criteria

Healthy blood donors visited the blood bank section of KFHU during the time of the study. Exclusion criteria: Blood donors who with comorbidities such as diabetes mellitus and hypertension.

### Study ethics

A written informed consent was obtained from all volunteer participant. The ethical approval for the study was issued by the ethical committee of the Institutional Review Board at Imam Abdulrahman Bin Faisal University (approval number: IRB-2019‐01‐300). All methods were carried out in accordance with the Helsinki declaration.

### Measuring sDPP4 level

**Fig 1:**
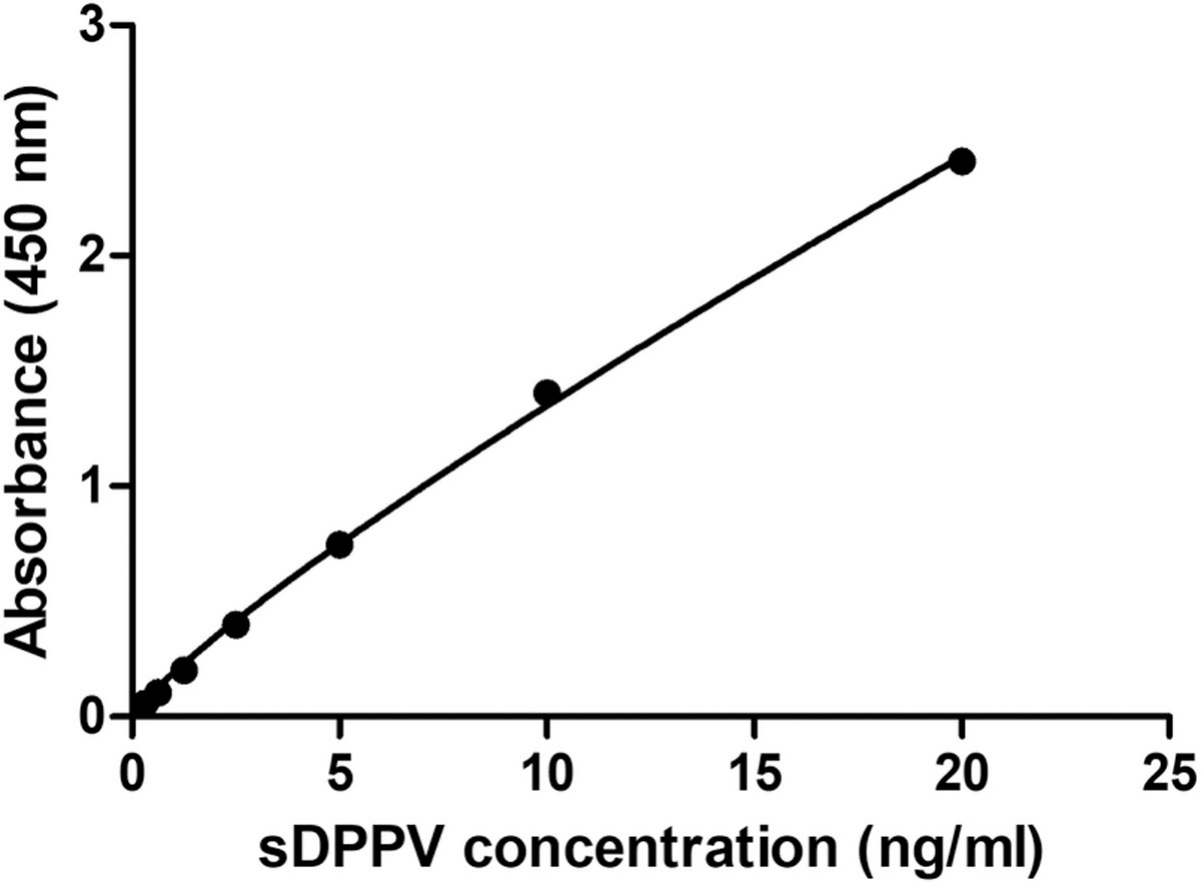
Standard curve for sDPP4 concentration. To determine the concentration of sDPP4 in the plasm samples, Graphpad Prism was used to fit a standard curve using nonlinear regression (curve fitting). The samples were diluted 100-fold before running the assay and the result was then multiplied by the dilution factor.

Plasma samples where centrifuged at 5000 rpm for 10 minutes before the test to precipitate cells if any. Ten microliters of the samples were added to the assay using the Quantikine ELISA kit (R&D Systems, Minneapolis, USA) for the quantification of the human DPP4 following the manufacturer’s instructions. Briefly, 100μL of assay diluent were added to each well. Then 50μL of the standards, control, or the 100-fold diluted samples were added per well. After 2 hours incubation, the well contents were aspirated and the well was washed 3 times with the provided washing buffer. Then, 200μL of the human DPPIV conjugate were added to each well and incubated for 2 hours at room temperature. After a three washing steps, 200μL of the substrate were added to each well and incubated for 30 minutes at room temperature. Lastly, 50μL of Stop Solution were added to each well. The optical density of each well was determined at 450nm with wavelength correction at 570 nm. Each sample was run in duplicate and a set of 8 standards were run in triplicate with each experiment. A standard curve was created by generating a four-parameter logistic curve-fit. Fig 1 shows a representative standard curve for sDPP4 concentration. To determine the concentration of sDPP4 in the plasm samples, GraphPad Prism was used to fit a standard curve using nonlinear regression (curve fitting). The samples were diluted 100-fold before running the assay and the result was then multiplied by the dilution factor. The mean minimum detectable dose (MDD) is 0.016 ng/ml as described in the kit. According to the manufacturer, no significant cross-reactivity or interference was observed with recombinant human ACE, ACE-2, DPP-6, ECE-1, ECE-2, Neprilysin, or recombinant mouse CD26.

### SNPs selection and genotyping

More than 18900 SNPs are located in the *DPP4* Homo sapiens gene according to the website of the National Center for Biotechnology Information (NCBI) (https://www.ncbi.nlm.nih.gov). These SNPs were filtered for those with minor allele frequency (MAF) of at least 0.05 and those affecting the transcript. Based on these filtering criteria, the following SNPs were selected for the study: rs35128070, rs1861978, rs79700168, and rs17574. DNA was extracted from the cellular fraction of the samples using the DNA mini kit following the manufacturer’s instruction procedures (Qiagen, Hilden, Germany). The TaqMan SNP genotyping Assay (Thermofisher Scientific, MA, USA) was used for the genotyping of the selected SNPs. The reaction was run on the Applied Biosystem QuantStudio™ 5-Realtime PCR system (Thermo Fisher Scientific, MA, USA). The software associated with the instrument was used for final allelic discrimination.

### Statistical analysis

All data were tabulated in Excel spread sheets. The frequencies, data grouping, means, and P value were calculated using the Statistical Package for the Social Sciences (SPSS) version 26. A P value of less than 0.05 was considered significant. GraphPad Prism software version 9.0 was used to draw scatter plots.

## Results

### Population description

A total of 419 volunteer blood donors were recruited to the study with average age 32.87 years (range 17–66 years). 219 participants were Saudi (average age 29.12 yeas) and 200 were from other ethnic groups (average age 36.98) including Arab ethnicity comprising participants from Jordan (39), Yemen (32), Egypt (23), Syria (12), Sudan (5), Palestine (5), Bahrain (2), Lebanon (3), and Iraq (2); Asian ethnicity comprising participants from India (46), Pakistan (12), Philippines (4), Bangladesh (3), Palau (2), and one from each of Taiwan and Sri Lanka. Additionally, two participants were from the United States of America, two from Canada, and one from each of Tanzania, Somalia, and Belgium. Female participants were 22 (5.3%) while male participants were dominant, n = 397 (94.7%). Table 1 describes the demographic data of the study population.

**Table 1:** Demographic data of the study population.

	Saudi-Arabs		Other-Arabs		Non-Arabs		total
	N	%	N	%	N	%	
**Gender**							
Females	19	86.4	1	4.5	2	9.1	22
Males	200	50.4	124	31.2	73	18.4	397
**Age**							
= <25	88	85.4	13	12.6	2	1.9	103
25-<35	77	53.1	44	30.3	24	16.6	145
35-<45	32	30.8	46	44.2	26	25.0	104
45-<55	20	35.1	20	35.1	17	29.8	57
= >55	2	20.0	2	20.0	6	60.0	10

### Soluble DPP4 level

**Fig 2:**
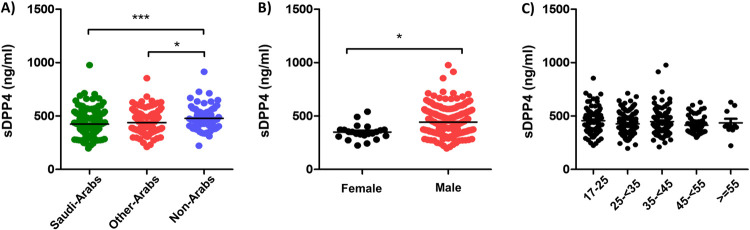
Average sDPP4 level among the study population according to (A) ethnicity, (B) Gender, and (C) age group.

The average level of serum DDP4 was significantly lower in Saudis (423.9 ng/ml) than other Arabs (438.5 ng/ml) and non-Arabs (478.7 ng/ml) (P value 0.0003 and 0.012, respectively) (Fig 2A). Similarly, the average level of sDPP4 was significantly lower among females (348.8 ng/ml) than males (443.0 ng/ml) (P value <0.001) (Fig 2B). There was no statistically significant difference in sDPP4 levels among different age groups of the study population (Fig 2C).

### SNP genotypes

**Fig 3:**
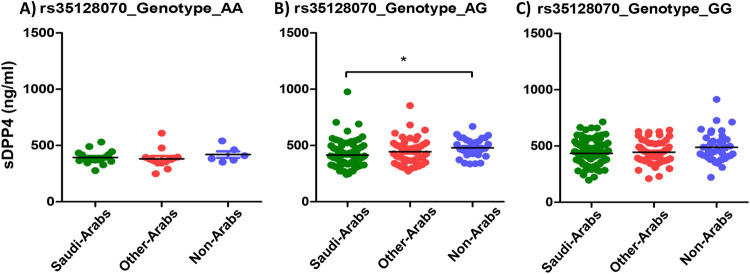
Average sDPP4 levels among different ethnic groups in relation to the rs35128070 genotypes.

**Fig 4:**
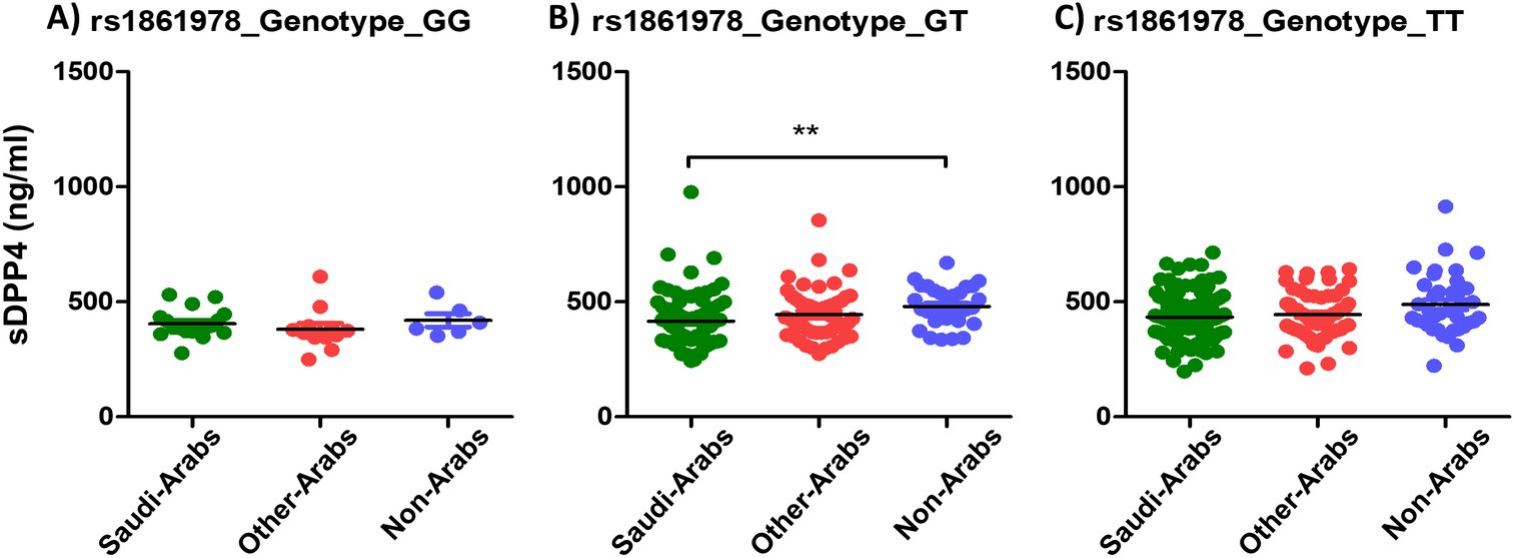
Average sDPP4 levels among different ethnic groups in relation to the rs1861978 genotypes.

Table 2 shows the frequency of the studied SNPs genotypes among the study population. The genotype AG of the SNP rs35128070 was significantly associated with lower sDPP4 among Saudis (414.8 ng/ml) compared to non-Arabs (479.4 ng/ml, P value 0.002) (Fig 3B). There was no statistically significant association between the AA or GG genotypes of the SNP rs35128070 and sDPP4 average level among the different ethnic groups (P value 0.480 and 0.113 respectively) (Fig 3A and 3C). Similarly, the genotype GT of the SNP rs1861978 was associated with lower sDPP4 among Saudis (415.3 ng/ml) compared to non-Arabs (479.3 ng/ml) (P value 0.002) but not the genotypes GG or TT (P value 0.298 and 0.084 respectively) (Fig 4).

**Table 2:** Frequency of SNPs genotypes among the study population.

	Saudi-Arabs		Other-Arabs		Non-Arabs		Total
	N	%	N	%	N	%	
rs35128070							
AA	18	48.6	13	35.1	6	16.2	37
GG	116	54.0	61	28.4	38	17.7	215
AG	83	50.3	51	30.9	31	18.8	165
rs1861978							
GG	18	50.0	12	33.3	6	16.7	36
TT	121	54.8	62	28.1	38	17.2	221
GT	79	49.1	51	31.7	31	19.3	161
rs79700168							
AA	66	55.5	36	30.3	17	14.3	119
GG	96	49.7	64	33.2	33	17.1	193
AG	57	53.3	25	23.4	25	23.4	107
rs17574							
AA	111	55.2	58	28.9	32	15.9	201
GG	23	48.9	16	34.0	8	17.0	47
AG	85	49.7	51	29.8	35	20.5	171

**Fig 5:**
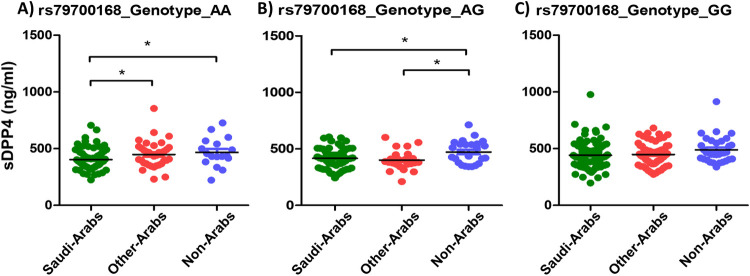
Average sDPP4 levels among different ethnic groups in relation to the rs79700168 genotypes.

The genotype AA of the SNP rs79700168 was associated with lower levels of sDPP4 among Saudis (403.7 ng/ml) compared to other-Arabs (448.2 ng/ml) and compared to non-Arabs (467.1 ng/ml) (P value 0.031 and 0.032, respectively). Additionally, the genotype AG of the SNP rs79700168 was associated with lower levels of sDPP4 among Saudis (417.2 ng/ml) and also among other-Arabs (400 ng/ml) compared to non-Arabs (472.2 ng/ml) (P value 0.015) but not the genotypes GG (P value 0.097) (Fig 5).

**Fig 6:**
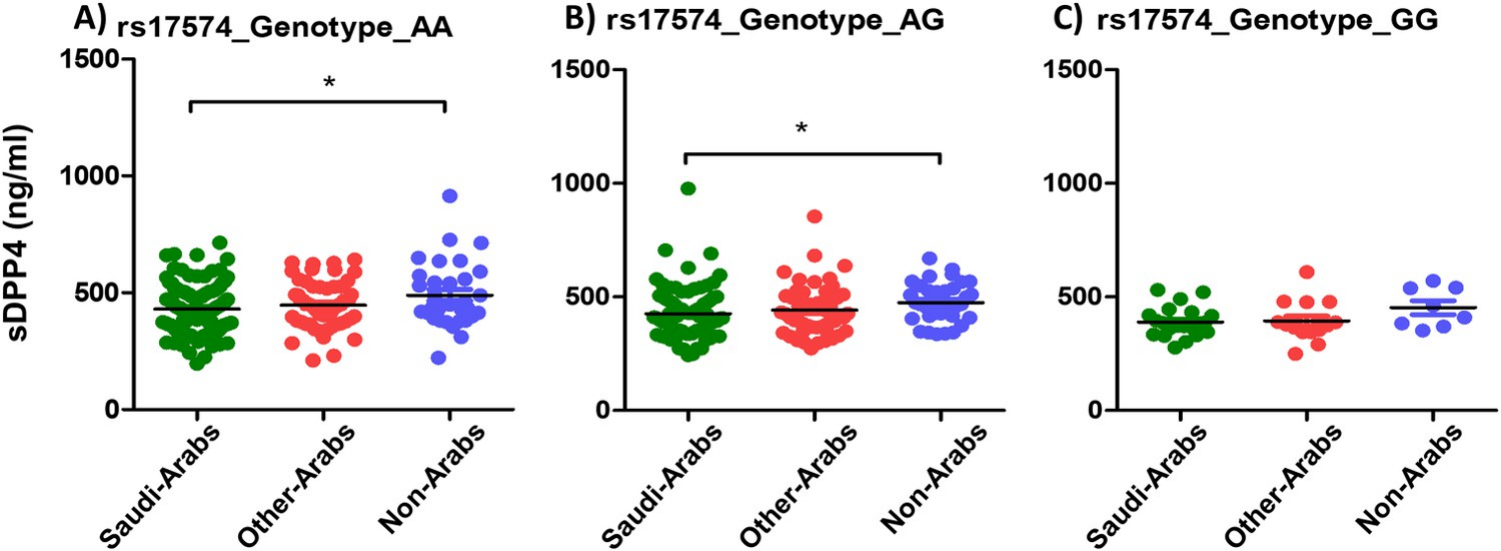
Average sDPP4 levels among different ethnic groups in relation to the rs17574 genotypes.

The genotypes AA and AG of the SNP rs17574 were both significantly associated with lower levels of sDPP4 among Saudis (430.3 and 425.1 ng/ml respectively) compared to non-Arabs (489.9 and 474.5 ng/ml respectively) (P value 0.027 and 0.014 respectively), while there was no statistical association between the genotype GG and serum levels of sDPP4 among the different ethnic groups (P value 0.118) (Fig 6).

## Discussion

DPP4 is described as a moonlighting protein for its multifaceted functions involving intracellular or extracellular functions depending on the expressing cells [33]. In addition to its multiple physiological roles, DPP4 was implicated in many pathological conditions including infection. Elevated levels of sDPP4 were found to be associated with poor treatment prognosis in chronically infected patients with hepatitis C virus genotype 1 [11, 12]. Additionally, high levels of sDPP4 were positively associated with progression to liver fibrosis and cirrhosis in hepatitis C virus infected patients [13]. The levels of sDPP4 were found to be intensely reduced during primary HIV infection and did not increase in response to combined anti-retroviral therapy [14]. These low sDPP4 levels were associated with rapid progression to AIDS [14]. Plasma levels of sDPP4 were also found to be reduced in patients hospitalized for severe COVID-19 infection [15]. Therefore, low level of sDPP4 may play a role in predisposing to infections caused by other pathogens.

Our results clearly demonstrate lower levels of sDPP4 among Saudi population compared to other ethnic groups, which may predispose the population to higher risk of acquiring MERS-CoV infection or increased disease severity, considering the role of DPP4 in immune response. Therefore, these results may indirectly explain the lengthy circulation of MERS-CoV among Saudi population. A previous study that assessed the levels of sDPP4 in MERS-CoV infected patients supports this finding [30]. The authors found that plasma and sputum levels of sDPP4 are reduced in MERS-CoV infected patients, and that sDPP4 can block MERS-CoV entry and reduce plaque formation experimentally [30]. Our conclusion is also consistent with another study that showed that high serum levels of sDPP4 were protective against MERS-CoV infection in human DPP4 transgenic mice, and were associated with attenuated morbidity and reduced mortality in infected mice [32]. Furthermore, the same study found that the use of recombinant sDPP4 significantly prevents MERS-CoV infection in the transgenic mice [32]. Similar observation has also been made in HIV-1 exposed but not infected female sex workers that their high blood concentrations of sDPP4 protected them from HIV infection [34]. Another study on the gene expression pattern in blood cells of HIV-1 highly exposed but seronegative female sex workers found that the most upregulated gene expression was of *DPP4* gene [35]. The authors suggested that sDPP4 cleaves the chemokine ligand (CCL) 5 protein, also known as RANTES, which in turn competes with HIV on CCR5 binding and reduces HIV infection [35].

The protective mechanism of higher sDPP4 level against MERS-CoV infection needs further exploration. Meta-analysis studies showed that the use of DPP4 inhibitor treatment significantly increases the incidence of upper respiratory tract and urinary tract infections [7, 31]. One hypothetical explanation is that the low sDPP4 level may reflect low sDPP4 level in sputum, which in turn reflects less virus-spike protein blocking activity in the lung. Moreover, it was shown that the level of sDPP4 is inversely correlated with the level of interleukin 10 (IL-10) and proportionally associated with the level of epidermal growth factor (EGF) in MERS-CoV infected individuals [30]. Therefore, high IL-10 (immune suppressor) levels and low EGF (immune enhancer) levels in case of low sDPP4 may have immune-suppressive effect on the lung epithelial mucosa. This hypothesis could be supported by the previous observation that all severe combined immunodeficiency patients and immunodeficient mice have reduced levels of sDPP4 [13]. Furthermore, additional evidence on the effect of low DPP4 levels on an overall reduced immune effector response is supported by the multiple DPP4 inhibitors approved for therapeutic use in different autoimmune diseases [36]. DPP4 inhibitors are used to treat autoimmune related diabetes mellitus in children and adults as well as autoimmune encephalomyelitis [37–39]. Certain DPP4 inhibitors are also used in the prophylaxis of acute Graft-versus host disease [40].

The females in our study had significantly lower levels of sDPP4 than males which has also been reported previously [41]. This could be attributed to the gender differences in normal body enzymatic levels [42]. However, the female population, especially the non-Saudi females, represented a minor fraction of the local blood donors included in the study, which may have influence our analysis.

There was no statistically significant difference in sDPP4 levels among different age groups of the study population. A previous study showed that the level of sDPP4 decreases in people older than 75 years compared to people younger than 75 years in a cohort of 52 individuals [43]. Another study showed that the levels of sDPP4 and its activity decreases with age in two groups of 40 and 20 individuals with mean age of 61±1.4 years and 60±2.0 years, respectively [44]. Our study population is larger in number and 97.6% of the population are younger than 55 years old. This might indicate that the effect of age on the sDPP4 levels is mainly apparent in older age groups.

We also investigated whether a genetic predisposing factor is behind the low levels of sDPP4 among the Saudi population. It was reported previously that SNPs in the *DPP4* may block MERS-CoV entry to cells [45]. Therefore, we selected SNPs in *DPP4* gene that have minor allele frequency of at least 0.05 to ensure proper penetration of the population. We then selected those SNPs that produce gene transcript variants. We found that the genotypes AG of the SNP rs35128070A>G, GT of the SNP rs1861978G>T, AA and AG of the SNP rs79700168G>A, and AA and AG of the SNP rs17574 were all significantly associated with lower average level of sDPP4 among Saudis compared to the other ethnic groups.

The SNP number rs35128070 is not studied previously and could produce upstream transcript variant according to the dbSNP database. The SNP number rs79700168 was picked up in a microarray scanning the *DPP4* gene in severely obese Caucasian individuals and was found not to be associated with cardiovascular disease in this cohort of individuals [46]. The SNPs number rs1861978 and rs17574 were included in a study investigating the *DPP4* gene polymorphism in Malaysian individuals with type 2 diabetes mellitus (T2DM), however, there was no association between these SNPs and T2DM [47]. The SNP number rs17574 was more intensely studied. In a cohort of Mexican individuals, rs17574 minor allele was associated with protection of hypoalphalipoproteinemia, while the GG genotype was associated with reduced levels of sDPP4 [48]. Another study from Mexico, however, did not find any association between rs17574 and COVID-19 disease severity nor sDPP4 serum levels [49]. In a group of Thai patients, who were chronically infected with hepatitis C virus genotype 1, rs17574 was not associated with sustained virological response nor sDPP4 serum levels [50]. Finally, rs17848915 (which was merged into rs17574) was found to be significantly associated with the methylation levels of *DPP4* promoter region, which negatively correlates with abundance of its mRNA in severely obese women with metabolic syndrome [51].

Despite these significant findings, the lower sDPP4 levels in the Saudi population compared to other ethnic groups could not be solely explained by these genetic variants in the *DPP4* gene because of its wide and diverse interaction partners and functions and multiple sources of expression, which may control its shedding. Body mass index (BMI) above 30 was previously reported to increase the sDPP4 levels. The BMI data were not available to us; therefore, we cannot make a conclusion about its effect in our study. However, a recent study reported that the prevalence of obesity (defined by BMI 30 and above) in the Eastern Province of the Saudi Arabia, which is the area of the current study, is 29.4% [52]. Hence, we do not expect the BMI to have a major effect on the reported low sDPP4 levels in our study. Although our study included a small sized population from the community, it found lower sDPP4 levels in the geographical area where most cases of MERS-CoV infections were reported. Future work should assess the levels of sDPP4 in exposed, uninfected individuals, like household contacts, in comparison with MERS-CoV infected patients to determine whether these findings will directly translate to susceptibility to infection by the novel virus.

## Conclusion

Herein, we report for the first time that Saudi population have lower levels of plasma sDPP4 than other ethnic groups, which was associated with genetic variants in the *DPP4* gene. We suggest that this may increase the susceptibility of the population to MERS-CoV infection and could be one of the reasons behind the long-lasting presence of the virus in the country.
